# Dosimetric Benefits of Omitting Primary Tumor Beds in Postoperative Radiotherapy After Transoral Robotic Surgery Using the Auto-Planning Technique

**DOI:** 10.7759/cureus.18065

**Published:** 2021-09-17

**Authors:** Taoran Cui, Matthew C Ward, Jeffrey A Kittel, Nikhil Joshi, Shlomo A Koyfman, Ping Xia

**Affiliations:** 1 Department of Radiation Oncology, Rutgers Cancer Institute of New Jersey, New Brunswick, USA; 2 Department of Radiation Oncology, Atrium Health, Charlotte, USA; 3 Department of Radiation Oncology, Aurora Health, Milwaukee, USA; 4 Department of Radiation Oncology, Cleveland Clinic, Cleveland, USA

**Keywords:** tumor bed sparing, adjuvant radiotherapy, head and neck cancer, auto planning, oropharyngeal squamous cell carcinoma

## Abstract

Introduction: It has been suggested that post-transoral robotic surgery (post-TORS) radiotherapy (RT) might reduce the dose to organs at risk (OARs) adjacent to the primary tumor bed; however, the evidence supporting this has yet to be sufficient. This study examined the radiation dose reduction to OARs by omitting the primary tumor bed through the use of an Auto-Planning (AP)-based workflow.

Methods: Twelve patients were identified who underwent post-TORS RT to the primary tumor bed and the unilateral/bilateral neck lymph nodes. In each patient, two treatment plans were designed: a Comprehensive (Comp)-plan treating the original planning target volume (PTV) including both the primary tumor bed and the lymph nodes, and a Neck-plan treating only the lymph nodes and omitting the primary tumor bed. Both plans were optimized using AP to ensure plan quality consistency. We compared the doses received by 95% of the primary tumor beds and lymph nodes (D95%) and our institutional dose constraints for the OARs between the Comp- and Neck-plans. Statistical analysis was performed using R Statistical Software (R Foundation for Statistical Computing, Vienna, Austria) with a two-tailed paired Wilcoxon signed-rank test.

Results: All plans met target dose coverage requirements with at least 95% of the PTVs covered with the corresponding prescription doses. The primary tumor bed in the Neck-plans was spared with a significantly lower mean D95% (25.9 Gy vs. 60.0 Gy; p < 0.01; Wilcoxon test). The mean dose to the oral cavity (20.9 Gy vs. 28.1 Gy; p < 0.01) and the supraglottis (36.9 Gy vs. 28.2 Gy; p < 0.01) was significantly lower in the Neck-plans.

Conclusion: This study suggests that sparing the primary tumor bed during post-TORS RT offers dosimetric benefits to nearby OARs with significant dose reductions to the oral cavity and supraglottis. Further study of the clinical risks and benefits afforded by this strategy is needed.

## Introduction

In recent years, the incidence of oropharyngeal squamous cell carcinoma (OPSCC) has increased, possibly as a result of human papillomavirus infection (HPV), and is estimated to double in the United States by 2030 [1]. Transoral robotic surgery (TORS) followed by postoperative radiotherapy (RT) or chemoradiotherapy (CRT) has become a standard treatment paradigm for patients with node-positive OPSCC [2,3]. With the primary tumors resected with TORS, it has been proposed that postoperative RT aims toward the neck lymph nodes while the primary tumor beds might be spared from postoperative RT if a negative margin resection is achieved, and the lower radiation dose to the primary tumor bed compared to the commonly used prescription dose (37 Gy vs. 60 Gy) is not associated with the increase in local failure [4,5]. As the volumes of treatment target decrease, the organs at risk (OARs) that are adjacent to the primary tumor beds, specifically the oral cavity, pharyngeal constrictor, supraglottis, and salivary glands, may receive reduced radiation dose. Dose reductions to these OARs would potentially reduce the incidences of radiation-induced toxicities, which becomes clinically beneficial in the era of de-intensification in HPV-positive OPSCC.

Inspired by recent studies to quantify the dose reduction to the OARs when excluding the primary tumor bed from the target volume, we adopted a comprehensive (Comp) method using the Auto-Planning (AP) technique to evaluate the potential dosimetric benefits of omitting the primary tumor bed from the target volume of the postoperative RT after TORS [6,7].

## Materials and methods

From an IRB-approved registry, patients diagnosed with HPV-positive pathological stage T1/2 base-of-tongue (BOT) OPSCC with at least one positive node who underwent TORS followed by postoperative RT between 2009 and 2015 at our institution (Cleveland Clinic, Cleveland) were retrospectively identified. The primary tumor bed and the ipsilateral neck lymph nodes were delineated as the high-risk clinical target volume (CTV), and the contralateral nodes as the low-risk CTV. The high- and low-risk planning target volumes (PTVs) were generated by adding a 2.5-mm uniform margin to the corresponding CTVs, and were originally treated with 60 Gy and 54-56 Gy, respectively, using simultaneous integrated boost RT. Each patient was then subsequently re-planned for two different treatment plans: one (Comp-plan) duplicating the original RT plan by treating the same comprehensive PTVs, and the other (Neck-plan) only treating the high- and low-risk neck lymph nodes with the aforementioned dose prescriptions, while the primary tumor bed was excluded from the original high-risk PTV.

The Comp- and Neck-plans were generated on a Varian TrueBeam linear accelerator (Varian Medical Systems, Palo Alto, CA, USA) with Millennium multi-leaf collimators (Varian Medical Systems). The volumetric modulated arc therapy (VMAT) technique with two 360° arcs of 6 MV photon beams was used in both plans. The Auto-Planning module in Pinnacle 9.10 (Philips Radiation Oncology Systems, Fitchburg, WI, USA) was used for plan optimization. The principles of AP have been detailed in previous publications [8,9]. In summary, in order to mimic the manual treatment planning process of an intensity-modulated RT (IMRT) plan, AP iteratively creates additional planning structures for an OAR, which are often called “ring” structures, based on the relative geometrical location of the OAR to PTVs. Based on the predefined optimization objectives and priorities of the OAR, AP first estimates the dose gradient between the PTV and the OAR, automatically assigns new optimization objectives and optimization weights to the new “ring” structures and performs the optimization to fine-tune the dose distribution (Table 1). Therefore, the AP technique is able to automatically optimize a plan to achieve reasonable OAR sparing while maintaining the dose coverage to PTVs with minimal human interventions.

**Table 1:** Auto-Planning optimization objectives used for the treatment plans DVH, dose-volume histogram; OAR, organ at risk The column of Compromise indicates whether the optimizer is allowed to compromise the DVH objective of the OAR to achieve better coverage to the PTV.

OAR	Constraints	Dose (Gy)	Volume (%)	Priority	Compromise
Brainstem	Max DVH	20	1	High	No
Cochlea	Max Dose	5		Medium	
Pharyngeal constrictor	Max DVH	56	50	High	
Pharyngeal constrictor	Mean dose	40			
Esophagus	Mean dose	20		Medium	
Larynx	Max DVH	56	5	Medium	
Larynx	Mean dose	30		High	
Mandible	Max DVH	70	1	High	
Oral cavity	Mean dose	30		High	
Contralateral parotid	Mean dose	25		High	
Spinal cord	Max dose	42		High	No
Contralateral submandibular	Mean dose	39		High	
Supraglottis	Mean dose	30		High	
Trachea	Mean dose	25		Medium	
Lips	Mean dose	20		Medium	
Lips	Max dose	35		High	
Normal tissue ring	Max dose	35		High	No

AP has been extensively used for various sites in our department with consistently reasonable results. The AP optimization objectives used in this study were determined based on our prior experience in planning and treating for OPSCC [10,11]. To achieve consistent dosimetric qualities, both plans were optimized with the same set of objectives without any manual adjustment to eliminate any planner bias. Because the anatomical structures, the prescription dose, and the optimization objectives were identical in both plans, except for whether or not the primary tumor was included in the high-risk PTV, the “ring” structures created by AP should be almost identical and assigned with similar optimization priorities. Therefore, the Comp- and Neck-plans generated by the AP technique should have comparable plan qualities. We should note that, despite being excluded from the high-risk PTV, the primary tumor bed was not considered as an avoidance structure during the optimization of the Neck-plans. After the AP optimization, both Comp- and Neck-plans were renormalized to ensure that at least 95% of the high-risk PTVs received 100% of the prescription dose (Table 1).

Following the institution protocol for OPSCC plans, the coverage to the primary tumor bed, the mean dose to the oral cavity, pharyngeal constrictor, supraglottis, larynx, ipsi-/contra-lateral parotid and submandibular, and the max dose to the mandible and the spinal cord were evaluated and compared between the Comp- and Neck-plans. Statistical analysis was performed using R Statistical Software (R Foundation for Statistical Computing, Vienna, Austria) with a two-tailed paired Wilcoxon signed-rank test. A p-value less than 0.01 was considered statistically significant.

## Results

Twelve patients with BOT OPSCC were included in this IRB-approved study. Of these, seven (58%) had T1 and five (42%) had T2 tumors. Six lesions were primarily left-sided and six were primarily right-sided. Bilateral neck-lymph nodes were treated in nine patients (75%) and three patients (25%) were treated unilaterally. Routinely, the CTV included anatomic levels II-IV of the neck, and in select cases included levels IB and V depending on tumor factors such as nodal burden or location of the primary. The contralateral neck was routinely treated for cancers with significant BOT involvement.

**Figure 1:**
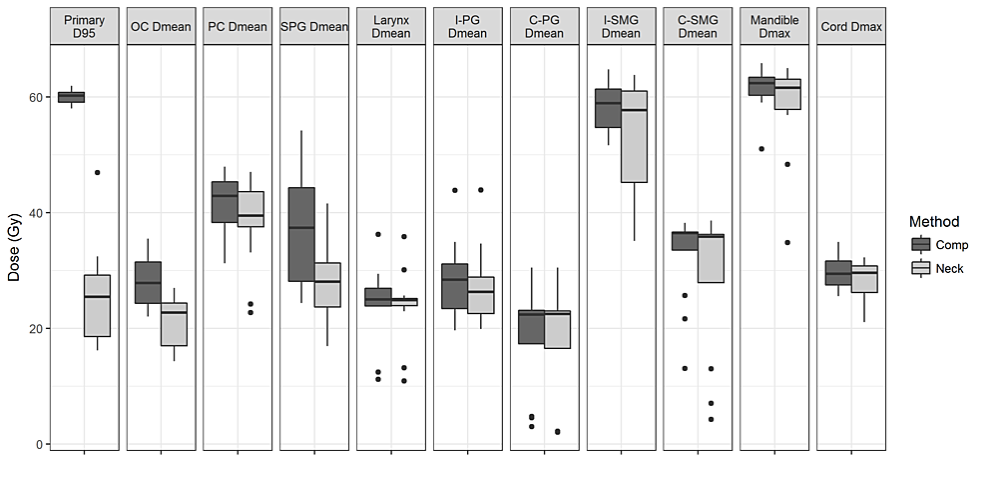
Boxplot of dosimetric endpoints between Comp- and Neck-plans for the 12 patients included in the study Primary, primary tumor bed; OC, oral cavity; SPG, supraglottis; PC, pharyngeal constrictor; I/C-PG, ipsi/contra-lateral parotid gland; I/C-SMG; ipsi/contra-lateral sub-mandibular gland

All Comp- and Neck-plans were successfully generated with the AP technique, and reviewed and approved by radiation oncologists. Dosimetric comparisons of the primary tumor beds and the OARs are summarized in Table 2 and Figure 1. The primary tumor beds were significantly spared in the Neck-plans with a reduced D95% (doses received by 95% of the primary tumor beds) of 25.9 Gy vs. 60.0 Gy in the Comp-plans (p < 0.001). The mean doses to the oral cavity and the supraglottis in the Neck-plans were also significantly lower than those in the Comp-plans, with 20.9 Gy vs. 28.1 Gy (p < 0.01) for the oral cavity and 28.2 Gy vs. 36.9 Gy (p < 0.05) for the supraglottis, respectively. Dose reductions were also found for all the other OARs in the Neck-plans, but none were statistically significant.

**Table 2:** Comparisons of the average dosimetric endpoints between Comp- and Neck-plans for the 12 patients included in the study

Structure	Constraint	Comp	Neck	p-value
Primary tumor bed	D95% (Gy)	60.0	25.9	<0.01
Primary tumor bed	V20Gy	100.00%	96.90%	<0.01
Primary tumor bed	V60Gy	94.80%	7.40%	<0.01
Oral cavity	Mean dose (Gy)	28.1	20.9	<0.01
Supraglottis	Mean dose (Gy)	36.9	28.2	<0.01
Contralateral parotid	Mean dose (Gy)	19.3	18.2	0.71
Contralateral submandibular	Mean dose (Gy)	32.7	29.1	0.22
Ipsilateral parotid	Mean dose (Gy)	28.7	27.5	0.48
Ipsilateral submandibular	Mean dose (Gy)	58.2	52.8	0.54
Mandible	D0.03cc (Gy)	61.4	58.3	0.38
Larynx	Mean dose (Gy)	24.3	23.9	0.67
Pharyngeal constrictor	Mean dose (Gy)	41.1	38.2	0.29
Spinal cord	D0.03cc (Gy)	29.6	28.3	0.59

**Figure 2:**
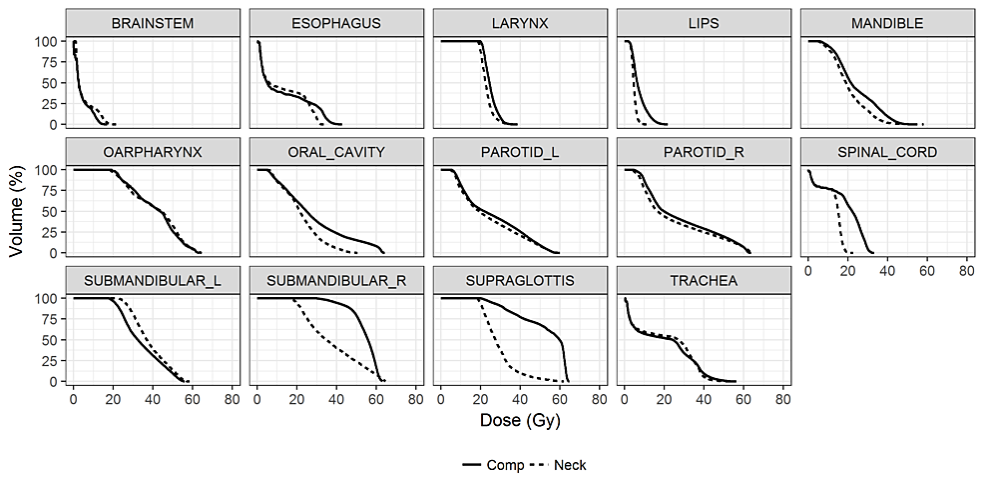
Comparison of dose volume histograms of organs at risk between the plan including the primary tumor bed as the PTV (Comp, in solid line) and the plan sparing the primary tumor bed (Neck, in dashed line) for a representative case PTV, planning target volume

**Figure 3:**
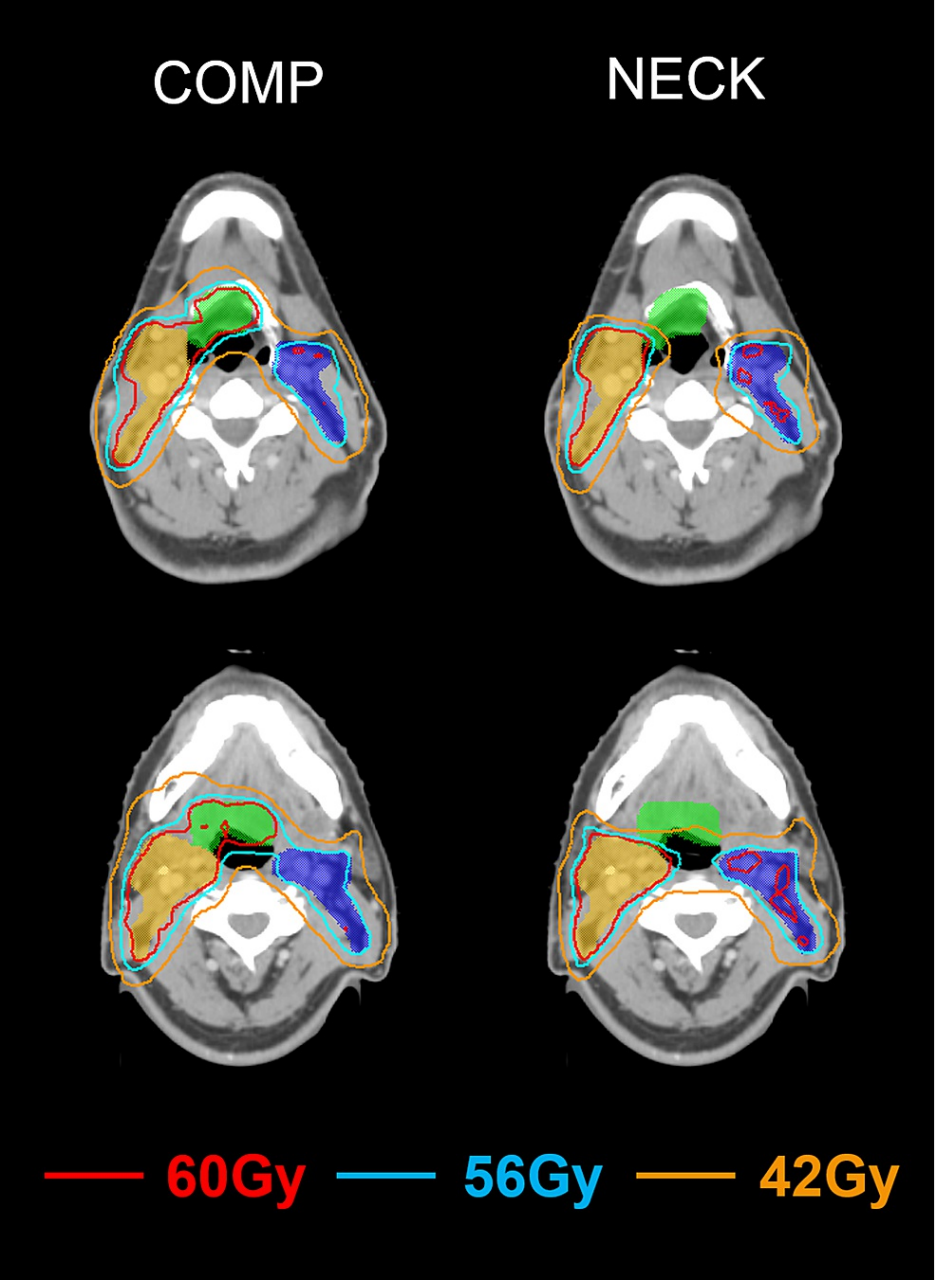
Comparison of isodose distributions (green shade, primary tumor bed; orange shade, high-risk PTV; blue shade, low-risk PTV) between the plan including the primary tumor bed as the PTV (left) and the plan sparing the primary tumor bed (right) for a representative case PTV, planning target volume

Figures 2, 3 demonstrate the comparison of the dose-volume histograms (DVHs) and the isodose distributions between the Comp- and Neck-plans for a representative case. In the Comp-plan, both the primary tumor bed and the ipsilateral nodes were covered by a 60 Gy isodose line. In the Neck-plan, however, the 60 Gy isodose line was conformed to the ipsilateral nodes only. Because the primary tumor bed abutted the supraglottis and the ipsilateral submandibular gland, sparing the primary tumor bed resulted in significant dose reductions in those two OARs. The other lateral OARs that were relatively distant from the primary tumor bed received comparable doses with the overlapping DVHs as shown in Figure 2, which also highlighted the similar dosimetric qualities of the two plans.

## Discussion

This study examined the dosimetric advantages of avoiding the primary tumor bed in postoperative RT for patients treated with TORS for their OPSCC. By excluding the primary tumor bed from the target volume, any OARs adjacent to the primary tumor bed received less radiation dose, which may reduce the likelihood of radiation-induced toxicities. Because of the large variations in plan qualities for head and neck radiotherapy planning, the extent of dose reductions in the OARs, as well as the primary tumor bed, have been inconsistently reported. In this study, we used a consistent planning methodology with the Auto-Planning technique to minimize the variations in plan quality. Despite not specifically considering the primary tumor bed as an avoidance structure, we spared the primary tumor bed significantly compared to the other studies [5-7].

Fried et al. first presented the concept of sparing the primary sites in their pilot study [6]. Possible dosimetric benefits were discussed for four patients treated with RT after transoral laser microsurgery for OPSCC. The dose reduction was only observed in the oral cavity, from the mean dose of 47.4 Gy when the primary tumor bed was included in the target volume to 22.3 Gy when the primary tumor bed was excluded, whereas the mean doses to contralateral parotid and pharyngeal constrictors were similar without a noticeable dose reduction. In their study, the primary tumor bed was considered as an avoidance structure in the primary-sparing plan and was therefore maximally spared with the mean dose decreasing from 61 to 42 Gy.

A more recent study by Lazarev et al. was conducted in a similar fashion but with a larger sample size of 21 patients [7]. This study reported the significant reduction in the mean dose of the oral cavity (29.8 Gy vs. 34.6 Gy) and the superior pharyngeal constrictors (42.9 Gy vs. 46.1 Gy) when omitting the primary tumor bed. Despite being excluded from the target volume, the primary tumor bed was not effectively spared in the primary-omitting plans. As a result, the mean dose of the primary tumor bed was 58.5 Gy, comparable to the prescription dose of 60 Gy to the tumor bed when treating both the primary site and the neck lymph nodes.

In this study, we presented a more comprehensive methodology to evaluate the dosimetric benefits of sparing the primary tumor bed. We used the AP technique to minimize the variations in the plan qualities. In theory, the dose distribution of a radiotherapy plan created using AP should solely depend on the anatomy of the plan, such as the location of the PTV and the relative geometries of an OAR to the PTV. Because of the identical anatomy between the Comp- and Neck-plans except for whether the primary tumor bed was included in the PTV or not, it was expected that any differences in the dose distribution between the two plans should only be found in the vicinity of the primary tumor bed, whereas the dose distribution far away from the primary tumor bed should be comparable. Therefore, by eliminating any potential differences in plan qualities, we were able to conduct valid comparisons to demonstrate the dosimetric benefits of sparing the primary tumor bed.

Notably, the dose to several OARs in our Comp- and Neck-plans were lower than those reported in the previous two studies, which also suggested better plan qualities achieved using the AP technique, as also observed in the previous literature [12]. While iteratively optimizing a plan, AP automatically creates multiple additional planning structures, and these planning structures not only fine-tune the dose distribution to better meet the objectives, but also help the optimizer to escape from local minima and, therefore, to achieve better plan quality.

In addition, dosimetric sparing of the primary tumor bed was verified in this study. The dose to the primary tumor bed significantly decreased in the Neck-plans (D95%, 60 Gy to 25.9 Gy; V60Gy, 94.8% to 7.4%). These dose reductions confirmed the purpose of excluding the primary tumor bed. It should be noted that, for the purpose of valid comparisons between the Comp- and Neck-plans, we did not intentionally minimize radiation dose to the primary tumor bed in Neck-plans, nor place any optimization objectives to the primary tumor bed. Instead, the same set of Auto-Planning optimization objectives was used in both the Comp- and Neck-plans to avoid any biased comparison.

Based on the significant dose reductions to the supraglottis and the oral cavity in the present study, it is expected that excluding the primary tumor bed during adjuvant RT after TORS would provide meaningful clinical benefits. It has been long observed and verified by multiple studies that the occurrence of radiation-induced dysphagia is strongly correlated with the mean dose to the supraglottis [13,14]. Moreover, the mean dose to the oral cavity has also been associated with the incidences of both mucositis and xerostomia [15,16]. Therefore, the dose reduction observed in the current study might be translated into decreased occurrences of radiation-induced toxicities. Future prospective investigations are warranted to validate these potential clinical benefits.

However, omitting the primary tumor bed from postoperative RT could also have negative consequences as it might also increase the risk of recurrence. Using the AP technique in our study, the mean doses to the primary tumor bed were approximately 30 Gy. This dose might be insufficient to treat residual cancer cells in the tumor bed. However, recently published studies on the omission of the primary tumor bed for patients with resected OPSCC reported higher doses to the primary tumor bed (40-43 Gy in [17] and 37 Gy in [4]) but with a favorable local control rate. These promising results suggested that this dose to the primary tumor bed might be able to effectively treat residual microscopic disease, especially for HPV-associated OPSCC.

## Conclusions

Excluding the primary tumor bed from the target volume of postoperative RT after margin negative TORS for OPSCC appears to be dosimetrically beneficial with a lower radiation dose to the oral cavity and supraglottis. These dose reductions could be potentially translated into a lower incidence of radiation-induced toxicities.
